# Everyday Functional Self-Awareness Patterns In Older People Living With HIV: A Multinomial Logistic Analysis

**DOI:** 10.1093/geroni/igaf122.2161

**Published:** 2025-12-31

**Authors:** David Vance, Michael Crowe, Victor Del Bene, Pariya Fazeli, Despina Stavrinos, Alexandra Jacob

**Affiliations:** University of Alabama at Birmingham, Birmingham, Alabama, United States; University of Alabama at Birmingham, Birmingham, Alabama, United States; The University of Alabama at Birmingham, Birmingham, Alabama, United States; University of Alabama at Birmingham, Birmingham, Alabama, United States; The University of Alabama, Tuscaloosa, Alabama, United States; University of Kentucky, Lexington, Kentucky, United States

## Abstract

Nearly 30% of people living with HIV (PLWH) have some mild-to-moderate cognitive impairment which can impact performance on instrumental activities of daily living (IADLs). Some PLWH also experience impaired self-awareness in their ability to perform IADLs. In fact, differences between self-reported and objectively measured IADL performance reveals some PLWH lack accurate awareness of functional abilities. This cross-sectional study examined self-awareness of everyday functioning in 260 middle-aged and older PLWH (40+ years) of whom most were African Americans (82.5%). Participants completed a comprehensive assessment, including cognitive performance testing and subjective and objective measures of everyday functioning. IADL domains, such as financial management, grocery shopping, medication management, and telephone use, were assessed by comparing self-reported performance on the Lawton and Brody IADL Questionnaire with objective performance from the Timed Instrumental Activities of Daily Living (TIADL) test. Three groups emerged based on differences between self-reported and objective performance for each IADL domain: accurate reporters, over-reporters (underestimating their abilities), and under-reporters (overestimating their abilities). Multinomial logistic regression models identified correlates of self-awareness for each IADL domain. Results indicated that under-reporting was more common than over-reporting, especially for grocery shopping (48.6%), medication management (23.2%), and telephone use (22.6%). Higher depressive symptoms predicted over-reporting in financial management and grocery shopping while lower executive function and word-reading scores predicted under-reporting. Men were more likely to under-report grocery shopping difficulties. Individuals who under-reported problems with IADLs may also under-utilize compensatory strategies. These results emphasize the importance of examining self-awareness to support functional independence, especially as PLWH age.

